# Analyzing the upscaling potential and geospatial siting of calcination-free calcium hydroxide production in the United States

**DOI:** 10.1016/j.heliyon.2024.e32426

**Published:** 2024-06-04

**Authors:** Sara Vallejo Castaño, Erika La Plante, Laurent Pilon, Gaurav Sant

**Affiliations:** a Laboratory for the Chemistry of Construction Materials (LC^2^), Department of Civil and Environmental Engineering, University of California, Los Angeles, CA 90095, USA; b Department of Mechanical and Aerospace Engineering, University of California, Los Angeles, CA 90095, USA; c Institute for Carbon Management, University of California, Los Angeles, CA 90095, USA; d Department of Materials Science and Engineering, University of Texas, Arlington, TX 76019, USA; e Center for Advanced Construction Materials, University of Texas at Arlington, TX 76019, USA; f Department of Materials Science and Engineering, University of California, Los Angeles, CA 90095, USA; g California Nanosystems Institute, University of California, Los Angeles, CA 90095, USA

## Abstract

This study evaluates the techno-economic feasibility and the embodied carbon dioxide intensity (eCI) of a novel process for producing nominally pure (>95 mass %) calcium hydroxide without the need for the thermal calcination of limestone. The process relies on the aqueous extraction of calcium from alkaline industrial wastes following which portlandite (Ca(OH)_2_: CH, a.k.a. slaked lime or hydrated lime) is precipitated by application of a waste-heat based thermal swing. This approach takes advantage of the temperature dependent solubility of CH at ambient pressure. We evaluated the feasibility of implementing this process in the U.S. based on the geospatial availability of waste heat and slags as a Ca-source. For the base case, the cost of production of “Low-Temperature Portlandite (LTP)” is 2-to-3 times that of traditional portlandite (∼$180/tonne). The main driver of cost is the electricity demand for reverse osmosis (RO) which is used to concentrate Ca-ions in solution, and the costs of membrane replacement. Our sensitivity analysis showed that parity with the cost of production of traditional portlandite is readily achievable by selecting membranes with better durability (i.e., better pH resistance) and flux (i.e., higher permeability) without sacrificing selectivity. Significantly, LTP features an eCI that is between 40%- and - 80 % lower than its traditional counterpart when electricity is sourced from natural gas combustion or wind power, respectively. Finally, our geospatial analysis reveals that there are three areas in the U.S. with the potential for implementation of industrial-scale facilities that could produce at least 50 tonnes of pure Ca(OH)_2_ per day, while achieving a production cost of ∼$270 per tonne of Ca(OH)_2_, owing to the proximity between slag feedstocks and waste heat sources.

## Highlights

• Low-Temperature Portlandite production cost is 2 times that of traditional portlandite.
• Embodied carbon dioxide intensity 40 %–80 % lower than traditional portlandite.
• Three areas in the U.S. have potential for implementation of industrial scale production.

## 1 Introduction

Portlandite or hydrated lime (Ca(OH)_2_) is an ubiquitous mineral that finds use in the production of paper, glass, sugar, and steel; as well as in construction, water treatment, and agricultural applications [[1], [2], [3], [4], [5]]. More recently, hydrated lime has been studied as a CO_2_ capture agent, as a binder for concrete production [6,7], and as a thermochemical energy storage agent in solar thermal power applications [8,9]. Despite the potential environmental benefits of using hydrated lime, its production via the traditional “thermal process” emits at ∼0.8 tonnes (t) of CO_2_ per t of Ca(OH)_2_ due to the calcination of limestone (CaCO_3_) at elevated temperatures (∼900 °C) [10]. As a result, with a global production of 420 million tonnes of quick- and hydrated-lime [11], the lime industry added ∼320 million t of CO_2_ to the atmosphere in 2020.

According to the European Union's Lime Association, 38 % of lime production is used in the steel industry [1]. Lime is used in electric arc and basic oxygen steelmaking furnaces to remove impurities from iron ores, which results in an alkaline byproduct called slag [[12], [13], [14], [15]]. The worldwide generation of iron and steel slag byproducts – with a CaO content ranging between 20-to-50 mass % [13,16] – was estimated to be ∼300 million tonnes in 2020 according to the United States Geological Survey (USGS) [17]. Although some types of slag find use as supplementary cementitious material [5], many other types – such as basic oxygen furnace (BOF) slag – are landfilled because of their high free lime content, which results in expansion and volumetric instability in concrete [12,[18], [19], [20]]. To reduce the CO_2_ footprint of Ca(OH)_2_, we previously demonstrated a low-temperature (≤100 °C) calcination-free route to produce Ca(OH)_2_ from steel slag at the bench- and pilot-scales [21]. Therein, an alkaline Ca-containing solution is obtained by dissolving slag in water. The solution is concentrated using reverse osmosis (RO) to reach the saturation point of Ca(OH)_2_ at room temperature and then heated to >65 °C to precipitate Ca(OH)_2_ [21,22]. Our pilot scale study demonstrated the continuous production of high purity (>95 mass %) Ca(OH)_2,_ and elucidated strategies to improve the efficiency and decrease the electricity consumption of the process by decreasing the slag's particle size for dissolution and increasing the precipitation temperature, respectively. The approach follows the principles of circular economy because it enables resource reuse and recovery from industrial byproducts [23] and has the potential to reduce not only the CO_2_ footprint of lime production [24] but also the environmental impacts of quarrying limestone [25] and landfilling slag.

The proposed LTP production approach could be implemented in industrial facilities with large availability of waste heat such as thermal power plants or steel plants where the slag is produced. Nevertheless, the electricity consumption, the availability of waste heat and slag, and the proximity between these two resources could be barriers to upscaling this process. Therefore, this study examines the upscaling potential of producing Ca(OH)_2_ from unused (i.e., crystalline, air-cooled) slags and compares its CO_2_ footprint to traditional Ca(OH)_2_ production. Specifically, the techno-economic feasibility and the environmental impact of producing portlandite from alkaline industrial wastes at the commercial scale using waste heat from thermal power plants, were assessed using AspenPlus©. Additionally, the production cost and the CO_2_ footprint of hydrated lime was evaluated as a function of relevant variables such as the cost and source of electricity and slag. Finally, we elucidate the locations in the U.S. that are suitable to implement such a process via a geospatial analysis that considers the location and availability of slag and waste heat feedstocks.

## 2 Methodology

### 2.1 Process description and modeling

**Fig. 1:**
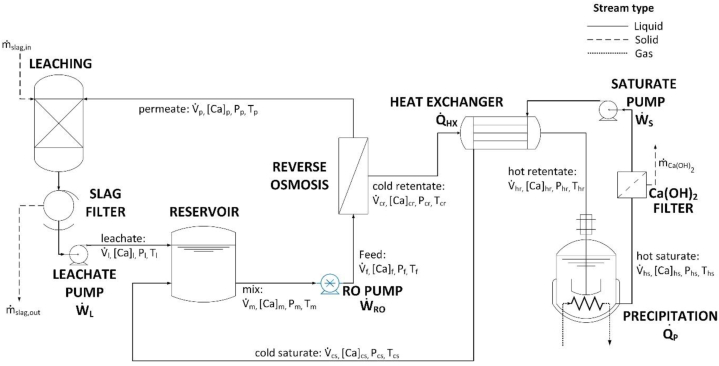
The system boundaries of the proposed Ca(OH)_2_ production process.

The proposed Ca(OH)_2_ production system was modeled using AspenPlus© [26] using data from our own bench- and pilot-scale experimentation and simulations [27]. Fig. 1 shows the process flow diagram (PFD) of the Ca(OH)_2_ production process. A base case that represents the production of ∼52 tonnes of Ca(OH)_2_ per day was selected as is typical for lime production [24]. The model evaluates the mass and energy balances of Ca(OH)_2_ production facility, and is used to estimate the capital and operating expenses of the process. Moreover, the model includes discrete process steps/unit operations including leaching, solid-liquid separations equipment, and a heat exchanger to recover the heat from the precipitation step and eliminate the need for cooling of the liquid reservoir.

*Leaching:* The process starts with the leaching of the slag in water in the leaching reactor. The water required for leaching is initially pristine, and thereafter sourced from recirculated permeate solution [27]. The leaching process was not simulated based on phenomenological equations because the rate and magnitude of calcium release from slag is influenced by many factors such as the type of slag, the particle size, the solid to liquid (s/l) mass fraction, and the type of leaching reactor (e.g., stirred tanks, fixed bed) [[28], [29], [30]]. Instead, the s/l mass fraction and the leachate concentration ${\left[Ca\right]}_{L}$ in the reactor were fixed as 0.01 and 8.1 mmol/L (mM), respectively, based on leaching experiments performed with a representative basic oxygen furnace (BOF) slag [27]. The model assumed that in time 100 mass % extraction of the calcium content of slag (20 mass% CaO based on XRF analysis [27]) to form Ca^2+^ ions is achieved. It is important to note that these leaching assumptions are optimistic and combine the best possible case scenario. After leaching, the resulting alkaline leachate is separated from the unreacted slag particles using a filter. The energy required for the solid-liquid separation was calculated as the energy required to pump the leachate through the filter using the leachate pump ${\dot{W}}_{L}$. In general, the pumping power was calculated with equation (1) as

(1) $${\dot{W}}_{i}=\frac{{\dot{V}}_{j}{\left(\Delta P\right)}_{i}\;}{\eta_{p}}$$

where $\eta_{p}=0.8$ is the hydraulic pump efficiency. ${\dot{V}}_{j}$ is the solution flow rate (in m^3^/s) of the stream *j* entering the pump, with j=l,m,s indicating leachate, mix, and saturate streams, respectively. ${\left(\Delta P\right)}_{i}$ indicates the pressure increase (in Pa) delivered by pump *I*, with i=L,RO,S indicating leachate, RO, and saturate pumps, respectively. The pressure increase delivered by the leachate pump was assumed to be ${\left(\Delta P\right)}_{L}=101,325\;Pa$:

*Reservoir:* The filtered leachate that is enriched in Ca-species enters the reservoir where it is mixed with the cool saturate stream. The latter is recirculated from the precipitation step to improve process efficiency. The calcium concentration, volumetric flow rate and temperature of the mixed stream exiting the reservoir were calculated with equations (2), (3), (4)) as

(2) $${\dot{V}}_{m}={\dot{V}}_{l}+{\dot{V}}_{cs}$$

(3) $${\dot{V}}_{m\;}{\left[Ca\right]}_{m}={\dot{V}}_{l}{\left[Ca\right]}_{l}+{\dot{V}}_{cs}{\left[Ca\right]}_{cs}$$

(4) $$T_{m}=\frac{{\dot{V}}_{l}T_{l}+{\dot{V}}_{cs}T_{cs}}{{\dot{V}}_{l}+{\dot{V}}_{cs}}$$

where $\dot{V},\left[Ca\right]\text{,}$ and T stand for volumetric flow rate, calcium concentration (in mol/m^3^), and temperature (in °C), respectively. The subscript *cs* corresponds to the cool saturate stream.

*Reverse Osmosis (RO) concentration:* To enable RO-based concentration, the mixed stream exiting the reservoir is pressurized using the RO pump from atmospheric pressure $P_{m}=101,325\;Pa$ to a feed pressure of $P_{f}=900,000\;Pa$. The pumping power was calculated using Equation (1) with ${\left(\Delta P\right)}_{RO}=798,675\;Pa$. The selection of the feed pressure was based on previous pilot-scale data [27]. To model the RO membrane separation step, a mass balance for the solution and for calcium species (Equations (5), (6))) was carried out as follows

(5) $${\dot{V}}_{f}={\dot{V}}_{p}+{\dot{V}}_{cr}$$

(6) $${\dot{V}}_{f\;}{\left[Ca\right]}_{f}={\dot{V}}_{p}{\left[Ca\right]}_{p}+{\dot{V}}_{cr}{\left[Ca\right]}_{cr}$$

where the subscripts f,p, and cr stand for feed, permeate, and cool retentate streams, respectively. The volumetric flow rate of the permeate stream was calculated with equation (7) as

(7) $${\dot{V}}_{p}=A_{RO}L_{p}\left({\Delta P}_{mem}-\Delta \pi \right)$$

where $A_{RO}$ is the RO membrane area, $L_{p}=8.64*10^{-12}m^{3}/\left(s{\;m}^{2}\;Pa\right)$ is the membrane permeability estimated from experimental data [21] and reported in literature [[31], [32], [33]]. ${\Delta P}_{mem}$ is the transmembrane pressure difference defined as the difference between the feed pressure $P_{f}$ and the permeate pressure $P_{p}=101,325\;Pa$, i.e., ${\Delta P}_{mem}=P_{f}-P_{p}$. The osmotic pressure difference between the feed and the permeate solutions was calculated with equation (8) as [34,35]

(8) $$\Delta \pi =R_{U}T_{f}\left[\left({\left[Ca\right]}_{f}+{\left[{OH}^{-}\right]}_{f}\right)-\left({\left[Ca\right]}_{p}+{\left[{OH}^{-}\right]}_{p}\right)\right]$$

where $R_{U}=8.314\;J\;{mol}^{-1}K^{-1}$ is the universal gas constant and $T_{f}$ is the feed temperature. Since the heat generated by the RO pump is negligible, the temperature of the feed is equal to the mix stream, i.e., $T_{f}$ = $T_{m}$. The concentration of $\left[{OH}^{-}\right]$ in all the streams was calculated by imposing electroneutrality in solution such that 2 $\left[{OH}^{-}\right]=\left[Ca\right]$. The permeate calcium concentration ${\left[Ca\right]}_{p}$ was calculated with equation (9) based on the membrane rejection coefficient R=0.99 according to membrane manufacturer data such that

(9) $${\left[Ca\right]}_{p}={\left[Ca\right]}_{f}\left(1-R\right)$$

*Retentate preheating and saturate recirculation*: Prior to precipitation, the cool retentate – concentrated using RO to the saturation point of Ca(OH)_2_ at room temperature – is preheated using the hot saturate stream exiting the precipitation reactor through the heat exchanger (Fig. 1). The heat absorbed by the cool retentate stream in the heat exchanger ${\dot{Q}}_{HX}$ was calculated with equatin 10 using the general energy balance [36]

(10) $${\dot{Q}}_{i}={\dot{V}}_{j}\rho c_{p}{\left(\Delta T\right)}_{j}$$

where ${\dot{V}}_{j}$ is the volumetric flow rate of the retentate stream exiting RO, $\rho =1000\;kg/m^{3}$ is the density and $c_{p}=4184\;J\;{kg}^{-1}K^{-1}$ is the heat capacity of the solution. ${\left(\Delta T\right)}_{j}=T_{j,out}-T_{j,in}$ indicates the temperature difference across the heat exchanger between the outlet and inlet streams. In the case of the recycle heat exchanger $T_{j,out}$ and $T_{j,in}$ correspond to the temperature of the hot retentate $T_{hr}$ and the cool retentate $T_{cr}$, respectively. Similarly, the heat delivered by the hot saturate stream was calculated with equation (10) with $T_{j,out}$ and $T_{j,in}$ equal to the temperature of the cool saturate $T_{cs}$ and hot saturate $T_{hs}$, respectively. The temperature of the cold saturate $T_{cs}$ was imposed at 30 °C to eliminate further cooling electricity requirements in the reservoir. The recycle heat exchanger surface area was estimated using equation (11) as

(11) $$A_{HX}=\frac{{\dot{Q}}_{HX}}{U\;LMTD}$$

where $U=849\;W/m^{2}K$ is the overall heat transfer coefficient [36], $A_{HX}$ is the area of the countercurrent heat exchanger and LMTD is the log mean temperature difference calculated with equation (12) as [36]

(12) $$LMTD=\frac{\left(T_{cs}-T_{cr}\right)-\left(T_{hs}-T_{hr}\right)}{\ln\left[\left(T_{cs}-T_{cr}\right)/\left(T_{hs}-T_{hr}\right)\right]}$$

**Fig. 2:**
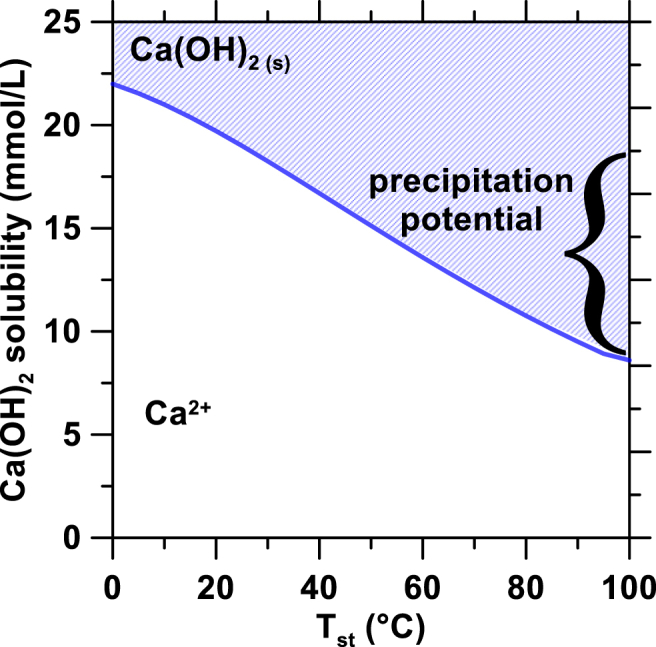
Ca(OH)_2_'s solubility as a function of temperature (AspenPlus database).

*Precipitation:* The hot retentate stream enters a continuously stirred-tank precipitation reactor. Within the precipitator, the solution was assumed to be in equilibrium with portlandite, i.e., ${Ca}^{2+}+2{OH}^{-}\;↔\;Ca{\left(OH\right)}_{2\;\left(s\right)}$. The concentration of the hot saturate stream ${\left[Ca\right]}_{hs}$ exiting the precipitation reactor was calculated based on the solubility of portlandite as a function of temperature ${\left[Ca\right]}_{hs}=f\left(T_{hs}\right)$ as illustrated in Fig. 2. The mass flow rate of solid Ca(OH)_2_ exiting the crystallizer ${\dot{m}}_{Ca{\left(OH\right)}_{2}}$ was calculated with equation (13) as

(13) $${\dot{m}}_{Ca{\left(OH\right)}_{2}}={\dot{V}}_{hs}\;M_{{Ca\left(OH\right)}_{2}}\left({\left[Ca\right]}_{cr}-{\left[Ca\right]}_{hs}\right)$$

where ${\dot{V}}_{hs}$ is the hot saturate volumetric flow rate, $M_{{Ca\left(OH\right)}_{2}}$ is the molar mass of Ca(OH)_2_ (in kg/mol) and ${\left[Ca\right]}_{cr}$ and ${\left[Ca\right]}_{hs}$ are the calcium concentration of the cool retentate and hot saturate streams, respectively. Additionally, since the mass of Ca(OH)_2_ precipitated is negligible compared to the volume of liquid (<0.1 mass %), the retentate and saturate flow rates were assumed to be equivalent such that ${\dot{V}}_{hr}={\dot{V}}_{hs}$. The heat required for precipitation is assumed to come from the flue gas exiting a thermochemical (e.g., natural gas, coal, etc.) power plant. The thermal power (in W) required for Ca(OH)_2_ precipitation ${\dot{Q}}_{P}$ was calculated using Equation (10) with $T_{j,out}$ and $T_{j,in}$ equal to the temperature of the hot saturate $T_{hs}$ and cool retentate $T_{hr}$, respectively. The temperature of the hot saturate $T_{hs}$ was set as 83 °C. After filtering out the precipitated Ca(OH)_2_, the hot saturated solution is recirculated using the saturate pump to the heat exchanger and thereafter to the reservoir, thereby completing the closed-loop process.

### 2.2 Analysis method

**Fig. 3:**
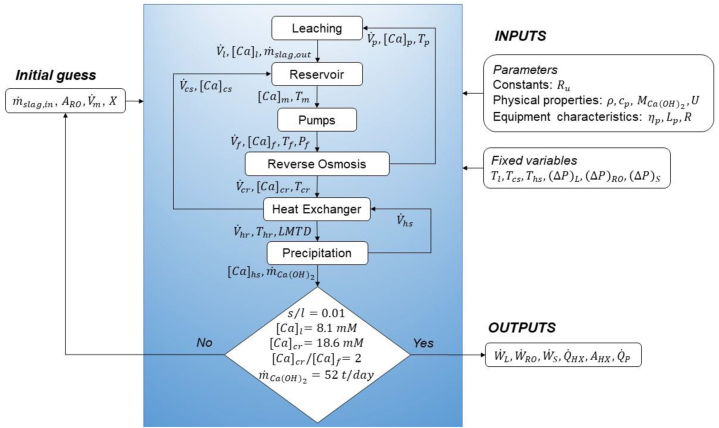
The block diagram of the method used to calculate the heat and power consumption of the LTP production process.

Fig. 3 shows a block diagram of the method to calculate the steady-state pumping power and the heating requirements to produce 52 tonnes per day of Ca(OH)_2_ via the LTP process. The variables used to initialize the solution were the inlet slag mass flow rate ${\dot{m}}_{slag,in}$, the leaching reaction conversion X, the RO membrane area $A_{RO}$, and the volumetric flow rate of the mix stream ${\dot{V}}_{m}$. After initialization, AspenPlus© solves equations (1), (2), (3), (4), (5), (6), (7), (8), (9), (10), (11), (12), (13)) simultaneously until convergence satisfies all the equations. The model satisfied the following constraints, which are representative of the operating conditions tested on the lab- and pilot-scale: (i) a s/l mass fraction in the leaching reactor of ∼0.01, (ii) a leachate Ca concentration of ∼8.1 mM (iii) a factor of 2 increase in the calcium concentration of the feed from the RO step (iv) a retentate concentration corresponding to the saturation point of Ca(OH)_2_ at room temperature, and (v) a Ca(OH)_2_ production rate ${\dot{m}}_{Ca{\left(OH\right)}_{2}}$ of ∼52 t/day.The initial guesses were modified iteratively until the solution of the model satisfied all the constraints.

### 2.3 Technoeconomic analysis (TEA)

*Capital expenditure (CapEx) estimation:* To estimate the cost of the RO step, the RO membrane area was calculated using WAVE® [37]. The simulation assumed single stage (single pass) concentration using FilmTec™ BW30-4040 (DuPont) spiral wound elements. The calcium concentrations and flow rates of the feed, permeate and retentate streams obtained from the AspenPlus© simulations were reproduced in WAVE® to estimate the membrane area required for RO considering constraints of the spiral wound elements such as minimum and maximum permeate and retentate flow rates and maximum pressure drop per element [37]. The membrane cost was assumed to be $19/m^2^ [[38], [39], [40]]. The total installed cost of the RO unit –including pressure vessels, pipes, support frame, etc. – was estimated based on a commercial RO plant [41] assuming that the cost of other items scales with the membrane cost (Fig. S1).

The capital costs of the leaching and precipitation reactors, the heat exchanger and the pumps were estimated using the AspenPlus Capital Cost Estimator™ (ACCE). The software sizes each unit operation and calculates equipment and installation costs encompassing equipment, instrumentation, civil work, electrical work, insulation needs and paint. Since the price basis considered was 2016, the price was escalated assuming an average inflation rate of 3.3 % per year up to 2022 [42]. The land cost was not considered because this process would be co-located alongside an existing power plant which typically has excess land available. Project contingency costs were not considered. Additionally, no discount rates, tax rates, or CO_2_ credits are considered in this analysis. The lifetime of the plant n was assumed to be 30 years. The distributed capital cost was calculated as the total capital expenditures, CapEx, divided by the plant lifetime n and the Ca(OH)_2_ production rate ${\dot{m}}_{Ca{\left(OH\right)}_{2}}$ (in tonnes/year), as shown in equation (14).

(14) $$distributed\;CapEx=\frac{CapEx}{n\;{\dot{m}}_{Ca{\left(OH\right)}_{2}}}$$

*Operating expenditure (OpEx)estimation:* The main operating costs considered were the costs of electricity, slag, slag transport, membrane replacement, and labor. The cost of electricity was calculated based on the (RO) pumping power requirements obtained from the AspenPlus© simulation, assuming the cost of electricity is $0.07/kWh [43]. The cost of slag was assumed to be $1 per t based on commercial price estimates for crystalline/air-cooled slags. A membrane replacement frequency of 5 years was assumed. Labor costs were assumed to be 2 % of the CapEx [41,44]. All the operating costs were divided by the Ca(OH)_2_ production rate assuming a plant capacity factor of 80 %. The cost of waste heat and water were not considered because the former is a co-product that is otherwise wasted, and water can be fully recirculated inside the process (i.e., water recovery greater than 99 % as demonstrated at the pilot-scale) [27].

### 2.4 Embodied carbon intensity (eCI)

**Fig. 4:**
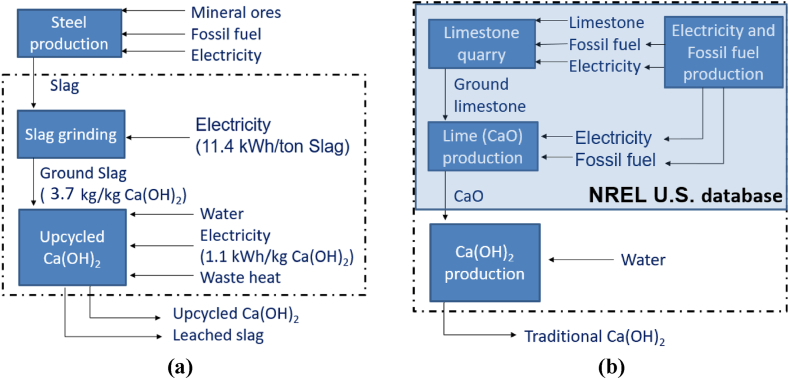
The cradle-to-gate LCA of **(a)** LTP and **(b)** traditional portlandite production processes. The dashed boxes indicate the boundaries of the LCA. The shaded box in traditional portlandite production indicates the data was retrieved from the NREL U.S. Database.

The goal of the eCI analysis was to evaluate material and process aspects of the LTP process as compared to *traditional portlandite* production. The life cycle impacts were modeled using the openLCA software [45], with the modified National Energy Technology Laboratory (NETL) CO_2_U LCI database [46] supplemented with the National Renewable Energy Laboratory (NREL) U.S. Life Cycle Inventory (USLCI) database [47]. The production, manufacture, and construction of manufacturing capital goods, capital infrastructure production equipment and vehicles, and personnel-related activities are excluded from the analysis. Fig. 4 shows the system boundary considered for the cradle-to-gate CO_2_ footprint analysis of the (a) LTP and (b) traditional portlandite production processes and describes key material/energy inputs and reference flows.

*Low-temperature portlandite production process:* The CO_2_ footprint of LTP production was calculated on the basis of 1 t of Ca(OH)_2_ produced via slag dissolution, RO concentration, and aqueous precipitation at a temperature of 83 °C. The electricity and material inputs were calculated from the base case scenario simulations. The specific energy consumption of grinding slag was calculated using equation (15) as [48].

(15) $$w_{g,slag}\left(\frac{kWh}{ton\;slag}\right)=W_{i}\left(\frac{10}{\sqrt{d}}-\frac{10}{\sqrt{d_{i}}}\right)$$

where $W_{i}=18.3\;kWh/ton\;slag$ is the Bond work index of slag [49], $d_{i}=9500\;\mu m$ is the initial particle size and d=100μm is the final particle size. The environmental impact of slag production was not considered because this material is a by-product of steel production. Following the same argument, no environmental impact was associated with the waste heat required for Ca(OH)_2_ precipitation. Three different sources of electricity generation were considered: (I) supercritical coal, (II) natural gas, and (III) solar thermal.

*Traditional portlandite production process:* The CO_2_ footprint of traditional portlandite production was calculated on the basis of 1 t of Ca(OH)_2_ produced via limestone quarrying, calcination, and lime (CaO) hydration. The electricity and fossil fuel consumption for limestone quarrying and calcination were taken from the NREL USLCI database [47]. It was assumed that limestone is quarried from open pits by blasting, followed by mechanical crushing and screening. Thereafter, limestone calcination in a rotary kiln – requiring electricity and fossil fuel – produces CaO. Finally, CaO hydration produces Ca(OH)_2_, but no energy consumption was associated with this final hydration step.

### 2.5 Geographic analysis of slag and waste heat availability in the U.S.

*Estimation of slag and CaO availability from steel production:* [50] The amount of slag produced in the U.S. was estimated from the CO_2_ emissions of steel production facilities assuming a ratio of CO_2_ emitted to steel produced of 0.64 [51], and a ratio of slag to steel produced of 0.12 [50,52]. The CO_2_ emissions from steel plants was obtained from the NATCARB database considering only the iron and steel production category [53].

*Estimation of waste heat from thermal power plants:* [54] Plant-level data for electricity generation, fuel consumption and cycle type reported in the Energy Information Administration (EIA) form EIA-923 was used to estimate waste heat generation [55]. The form reports the annual electricity generation $W_{elec}$ and the total heat input from the utilized fuel $Q_{fuel}$ for every thermal power plant in the U.S. The types of fuel considered for the geospatial analysis were coal and natural gas, operating with steam or gas turbines. Equations (16), (17)) were used to calculate the cycle efficiency and the waste heat for each plant [54].

(16) $$\eta_{cycle}=\frac{W_{elec}/\eta_{turbine}}{Q_{fuel}}$$

(17) $$Q_{waste}=Q_{fuel}-\frac{W_{elec}}{\eta_{turbine}}$$

where $\eta_{turbine}$ is the turbine efficiency taken as 86 % and 93 % for steam and gas turbines, respectively [54]. To validate the results, the cycle efficiency was compared to typical efficiencies of the Brayton and Rankine cycles.

*Relative distance between thermal power plants and slag facilities:* The locations of slag facilities were obtained from the National Slag Association. The location of the thermal power plants was obtained from form EIA-860 [56]. The distance from each power plant to every slag facility in the same state was calculated with equation (18) as

(18) $$D=\sqrt{{\left(K_{1}\Delta \varphi \right)}^{2}+{\left(K_{2}\Delta \lambda \right)}^{2}}$$

where Δφ is the latitude difference and Δλ is the longitude difference between the two points. $K_{1}$ and $K_{2}$ are constants. A MATLAB [57] script was developed to identify the closest slag facility to each power plant. The location data obtained was visualized using the QGIS software [58].

## 3 Results and discussion

### 3.1 Base case scenario

Table 1 summarizes the main results of electricity and heat consumption for the base case scenario. The total electricity consumption of the process is ∼1095 kWh per t Ca(OH)_2_ and it was dominated by the RO pumping demand. The total heat required for precipitation (${\dot{Q}}_{P}+{\dot{Q}}_{HX}$) is ∼113725 kWh/t Ca(OH)_2_, i.e., two orders of magnitude larger than the total electricity consumption. However, at steady state 90 % of this heat is supplied through heat recovery using a heat exchanger. Hence, for the production rate considered herein, the heating rate required at steady state to produce 52 t per day of Ca(OH)_2_ is ∼26 MW, which is compatible with the residual heat flux of median-size thermal power plants in the U.S [54]. The results demonstrate that the heat exchanger is essential because it reduces the waste heat input required at steady state by an order of magnitude by preheating the cold retentate. Second, it cools down the hot saturate stream to 30 °C which is required for continuous operations. If electricity was necessary for cooling the feed stream, the operating costs of the process would be prohibitive.

**Table 1:** A summary of the AspenPlus© simulation of the base case scenario to produce ∼52 tonnes of Ca(OH)_2_ at a single plant location per day.

Base Case Scenario	
**Ca(OH)**_**2**_**production rate**${\dot{m}}_{Ca{\left(OH\right)}_{2}}$ from simulation	52.4 t Ca(OH)_2_/day
**Ca(OH)**_**2**_**production rate**${\dot{m}}_{Ca{\left(OH\right)}_{2}}$**considering capacity factor**	41.9 t Ca(OH)_2_/day
**RO pump energy consumption**${\dot{W}}_{RO}$	971.5 kWh/t Ca(OH)_2_
**RO membrane area**$A_{RO}$	169,232 m^2^
**Low-pressure pumps energy consumption**${\dot{W}}_{L}+{\dot{W}}_{S}$	123.2 kWh/t Ca(OH)_2_
**Slag consumption**${\dot{m}}_{slag,in}$	3.7 t slag per t Ca(OH)_2_
**Precipitation heat**${\dot{Q}}_{P}$	11,818 kWh/t Ca(OH)_2_
**Heat recovery from heat exchanger**${\dot{Q}}_{HX}$	101,906 kWh/t Ca(OH)_2_
**Heat exchanger area**$A_{HX}$	52,321 m^2^
**Feed flow rate**	3499.7 m^3^/t Ca(OH)_2_

### 3.2 Technoeconomic analysis

**Fig. 5:**
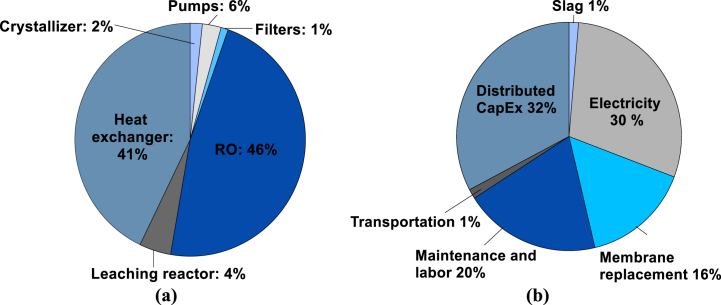
The breakdown of: **(a)** capital costs and **(b)** fully loaded production costs of a commercial scale LTP production plant.

The total capital expenditure of a 52 t per day LTP plant was estimated to be $40,827,448. Fig. 5 shows the breakdown of (a) the capital and (b) the fully loaded Ca(OH)_2_ production costs for the base case scenario. Fig. 5(a) shows that the RO unit and the heat exchanger account for 87 % of the total upfront investment due to the large membrane and heat exchange areas required for the concentration and heat recovery processes, respectively. Based on the membrane area requirement (Table 1) the cost of membranes was estimated as $3,230,406. Thus, capital expenditures for the fully installed RO unit were estimated to be $16,475,069, i.e., ∼5.1 times the cost of membranes. The assumptions considered to calculate the capital cost of the RO unit – including membranes, pressure vessels and piping – are presented in Fig. S1 [41]. Fig. 5(b) shows that slag and transportation are the least expensive operating costs. Moreover, the analysis indicates that the main driver of the operating cost was the RO step, due to electricity and membrane replacement costs.

Table 2 shows the operating cost breakdown of the base case scenario. The total operating expenses were calculated to be $182.92 per t Ca(OH)_2_. The fully loaded production cost – also known as the break-even point – calculated as the sum of *OpEx* plus *Distributed CapEx* for a plant producing 52 tons of Ca(OH)_2_ per day was $271.86 per ton Ca(OH)_2_. The distributed capital cost calculated with Eq. (14) was $88.94 per ton Ca(OH)_2_. The fully loaded production cost of the LTP production process is ∼2–3 times higher than the wholesale price of Ca(OH)_2_ – ranging between $160 to $180 per t Ca(OH)_2_ based on manufacturer data – but could compete in the specialty market where the fine size, and controlled shape of similar products ranges between $460 and $560 per t Ca(OH)_2_. Assuming a midpoint sale price of $510/t Ca(OH)_2_, the margin would be 47 % and the payback period would be 8.1 years.

**Table 2:** The detailed breakdown of costs for the base case scenario.

Cost variables	Assumption	Cost ($/t Ca(OH)_2_)
**Direct labor**	2 % of capital cost per year	53.37
**Slag**	$1 per ton of slag	3.7
**Electricity**	$0.07 per kWh	67.93 (RO pump)
		8.62 (Low pressure pumps)
		3.37 (Grinding)
**Membrane replacement**	Every 5 years	42.23
**Slag transport**	20 miles ($0.051 per ton-mile)	3.7
**OpEx**		182.92
**Distributed CapEx**	30-year plant lifetime	88.94
**Fully loaded production cost**		271.86

**Fig. 6:**
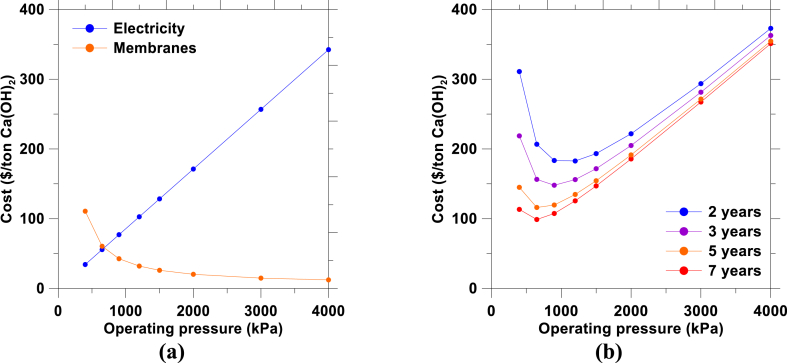
(a) The cost of electricity and membrane replacement (assuming membranes should be replaced every 5 years) as a function of feed pressure for RO concentration, and (b) A sensitivity analysis of optimum combined operating cost of RO (electricity + membrane replacement) as a function of the membrane replacement frequency.

First, a sensitivity analysis was performed with particular emphasis on the RO step, since it represents the largest cost fraction of Ca(OH)_2_ production. Fig. 6 shows the operating cost of RO combining electricity and membrane replacement per t of Ca(OH)_2_ as a function of feed pressure for the following scenarios: (a) assuming membranes are replaced every 5 years (base case scenario) and (b) for different membrane replacement frequencies. Fig. 6(a) shows that the minimum combined RO operating pressure for the base case scenario is around 650 kPa. Additionally, it demonstrates there is a tradeoff between electricity and membrane cost as a function of RO feed pressure. Fig. 6(b) demonstrates that a lower membrane replacement frequency would reduce by half the RO operating cost. Moreover, the optimum operating pressure decreased with decreasing membrane replacement frequency. The results indicate that durable RO membranes that can withstand high pH conditions would make the LTP process more economical. And, although some RO membranes claim a pH operating range between 1 and 13, the extent to which operating under high pH conditions would decrease the membrane lifetime remains unclear.

**Fig. 7:**
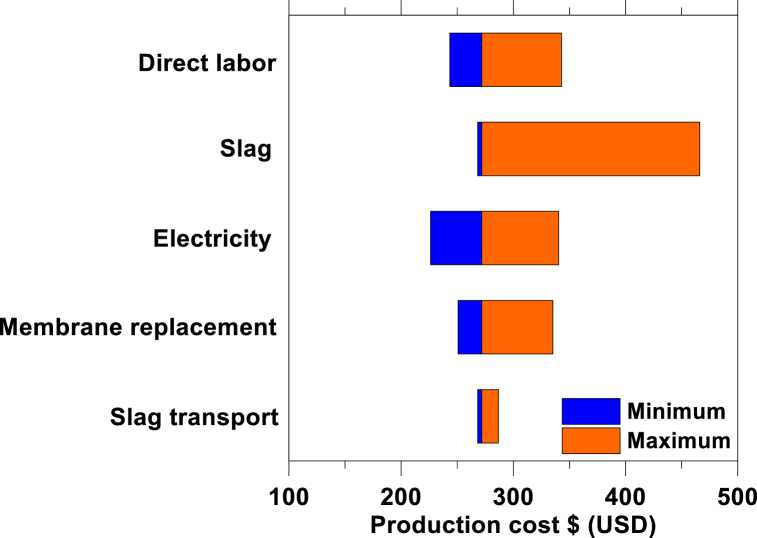
A sensitivity analysis showing the fully loaded production cost of Ca(OH)_2_ including the cost of labor, slag, electricity, transport, and membrane replacement frequency.

Second, a sensitivity analysis on the operating cost was performed considering the variables and ranges therein shown in Table 3. Fig. 7 shows the results of the sensitivity analysis. The model is particularly sensitive to the cost of slag especially if the slag's Ca-content is not effectively utilized because of the large amount of slag required to produce 1 t of Ca(OH)_2_. A larger Ca-extraction efficiency would reduce the sensitivity of the model to the cost of slag, underscoring the importance of maximizing calcium extraction during leaching, which if unoptimized is only ∼10 mass % [21]. The frequency of membrane replacement is the second variable that strongly influences the production cost of Ca(OH)_2_. According to the membrane manufacturer, the replacement frequency can be as large as every 5–7 years. However, since the concentration process operates at a pH higher than the recommended by the manufacturer, the long-term stability of the membranes may be compromised due to membrane fouling and degradation. Finally, the cost of slag transport highlights that the source of slag should be as close as possible to the Ca(OH)_2_ production plant. The minimum and maximum production costs that could be achieved combining all the variables considered in the sensitivity analysis range between ∼$80 and $595 per t Ca(OH)_2_.

**Table 3:** The range of the primary variables that affect the cost of LTP production.

*OpEx* Variables	Minimum	Maximum	Units
**Direct labor**	2 %	8 %	% of CapEx per year
**Slag cost**	$0	$10	per ton of slag
**Electricity**	$0.03	$0.13	per kWh
**Membrane replacement**	1 year	5 years	Replacement frequency
**Slag transport**	1 mile	100 mile	Transport distance

### 3.3 Lifecycle analysis (LCA)

**Fig. 8:**
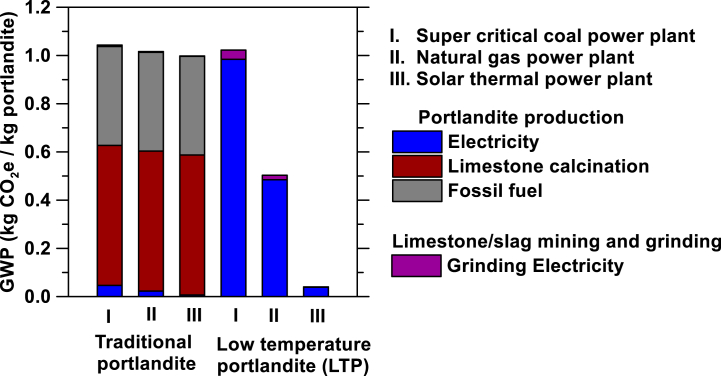
The 100-year global warming potential (GWP) of traditional and LTP production assuming three different sources of electricity: (I) Coal power, (II) Natural gas power, and (III) solar thermal power.

Fig. 8 shows the 100-year global warming potential (GWP) of traditional and low-temperature Ca(OH)_2_ production assuming 3 different sources of electricity: (I) coal power, (II) natural gas power, and (III) solar thermal power. The figure shows that the traditional route to produce portlandite consistently generates more CO_2_ than the low-temperature route. The main source of CO_2_ emissions is limestone's calcination for the traditional route, and electricity for the alternate route. In general, low temperature portlandite features a CO_2_ footprint that is 40 %–80 % lower than the traditional product when electricity is sourced from natural gas or renewable power, respectively. As such, when the source of electricity produces less CO_2_ (i.e., the grid emissions factor, *g*_*f*_, kgCO_2_/kWh of electricity) the environmental impact of LTP decreases. Since the LTP process requires large amount of low-grade heat, beyond power generation, such heat could be sourced from industrial processes (e.g., steel production), or solar thermal or geothermal heat. The advantage of the proposed route becomes evident from Fig. 8. For the traditional production process, even if fossil fuels were displaced by a renewable energy source like hydrogen the CO_2_ emissions would still be significant due to the process emissions associated with the calcination of limestone. It is important to minimize the eCI of portlandite considering that 1 kg of Ca(OH)_2_ can absorb up to 0.59 kg of CO_2_. This, Ca(OH)_2_ produced using the LTP process and renewable energy could produce a material that absorbs more CO_2_ than what is required for its production throughout its life cycle, i.e., a CO_2_-negative material.

### 3.4 Geospatial distribution analysis

**Fig. 9:**
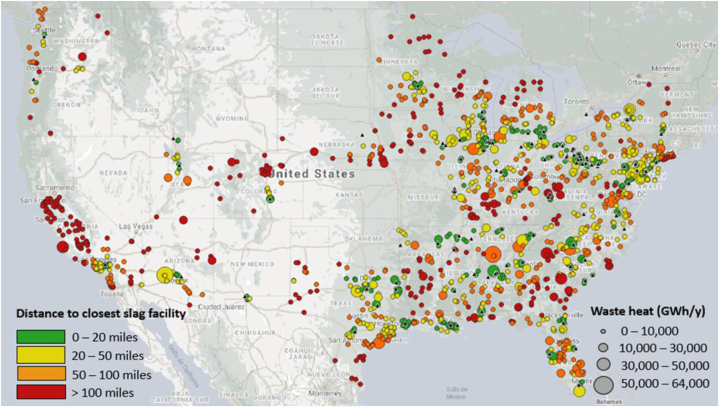
The geospatial availability of iron and steel slag (black triangles) [61] and waste heat (circles) [55,56] in the continental U.S.

As a starting point, U.S. thermal power plants were categorized based on their yearly waste heat generation and their distance to the closest slag facility. Fig. 9 shows the geospatial availability of iron and steel slags and waste heat sources. More than 50 % of the total slag produced is in the Midwest region of the U.S. The amount of slag produced per year in the U.S. was calculated to be ∼10.3 million tons per year, in general agreement with other data sources [59]. For example, the USGS estimated that 14 million tons of slag are produced per year in the U.S [60], suggesting that the present calculation underestimates domestic slag production. The results also highlight the need for more updated CO_2_ emissions accounting in the steel industry since the newest database available was from 2015 [53]. But, as a rough estimate, if 40 mass % of the slag was composed of CaO, the domestic production of Ca(OH)_2_ from slag would be ∼4.1 million tons per year, indicating that the production of Ca(OH)_2_ from slag could replace at most, a quarter of the entire lime market in the U.S. albeit while greatly reducing its carbon footprint.

Fig. 9 shows waste heat sources categorized based on the nominal capacity of the power plant (circle size) and by the distance to a slag source (circle color). Hence, large green circles represent the best implementation sites for the technology, whereas small red circles represent the least feasible sites. The figure indicates that there are at least 19 thermal power plants that generate between 10,000 and 50,000 GWh/y of waste heat located within 20 miles of a slag facility, and 41 thermal power plants with the same waste heat generation capacity located within 20 and 50 miles of a slag facility. Based on this assessment, there is substantial potential for LTP production in the Northeast, Southeast and Midwest regions of the U.S. Based on the number of slag facilities and thermal power plant generating more than 10,000 GWh/y, the states with more potential for implementing such projects are Pennsylvania, Alabama, Texas, Louisiana, Florida, North and South Carolina. Other states that have many slag facilities are Ohio, Illinois, and Indiana. Individual power plants in these states generate less than 10,000 GWh/y, but collectively they provide enough waste heat to process all the slag generated in these states. Moreover, the slag could also be treated using the waste heat generated in the steel production process (10 GJ/t crude steel) [62]. This study indicates that there is more than sufficient waste heat to produce Ca(OH)_2_ using the LTP process and that the limiting resource is in fact slag. This suggests that identifying alternate sources of Ca-bearing alkalinity would be an important consideration for future studies.

## 4 Summary and conclusions

This study evaluates the techno-economic feasibility and the environmental impact of a novel process for the low-temperature production of Ca(OH)_2_ using iron and steel slags, and waste heat. We analyzed the heat, mass, and energy balances for a base case plant that implies the production 52 tonnes per day of Ca(OH)_2_. It is determined that the cost of production ranges between $80 and $560 per t of Ca(OH)_2_, with the base case scenario yielding a production cost of $271 per t of Ca(OH)_2_; i.e., 2–3 times higher than current wholesale prices. The main drivers of the cost are the electricity, membrane replacement, and distributed capital costs. Nevertheless, the sensitivity analysis showed that a competitive operating cost can be achieved by increasing the membrane lifetime and decreasing the electricity consumption, which could be readily achieved using membranes with higher pH resistance and that operated at lower pressure. In addition, there is a need to improve the rate and extent of Ca-extraction during leaching, e.g., by using packed bed reactors, to maximize the types of slags that can be utilized, and to minimize slag consumption without increasing the electricity consumption. The proposed Ca(OH)_2_ production route features a CO_2_ footprint that is 40 %–80 % lower than traditionally (“thermally”) produced Ca(OH)_2_ when electricity is sourced from natural gas or wind power, respectively. Finally, a geospatial analysis elucidates that, due to the proximity between feedstocks and waste heat sources, there are ∼60 thermal power plants in the U.S. with the potential to up-scale and implement this technology at an industrial-scale today.

## Data availability Statement

Data will be made available on request.

## CRediT authorship contribution statement

**Sara Vallejo Castaño:** Writing – review & editing, Writing – original draft, Visualization, Validation, Methodology, Investigation, Formal analysis, Data curation, Conceptualization. **Erika La Plante:** Writing – review & editing, Supervision, Software, Resources, Project administration, Methodology, Conceptualization. **Laurent Pilon:** Supervision, Methodology, Investigation. **Gaurav Sant:** Writing – review & editing, Supervision, Investigation, Funding acquisition, Conceptualization.

## Declaration of competing interest

The authors declare the following financial interests/personal relationships which may be considered as potential competing interests:Gaurav Sant reports financial support was provided by 10.13039/100013165National Energy Technology Laboratory. Laurent Pilon reports financial support was provided by 10.13039/100000001National Science Foundation. Gaurav Sant reports was provided by The Chan Zuckerberg Initiative. Gaurav Sant reports was provided by National Institute of Standards and Technology. Gaurav Sant has patent #US20210024364A1 issued to University of California. The authors acknowledge financial support for this research from the 10.13039/100000015Department of Energy via the: U.S. Department of Energy's 10.13039/100006120Office of Fossil Energy and Carbon Management's 10.13039/100013165National Energy Technology Laboratory (NETL: DE- FE0029825), TRANSCEND: a UCLA-10.13039/100000161NIST Consortium that is supported by its Industry and Agency partners, The Chan Zuckerberg Initiative (10.13039/100014989CZI), 10.13039/100000001National Science Foundation NRT-INFEWS: Integrated Urban Solutions for Food, Energy, and Water Management (Grant No. DGE-1735325), and Shell India.

## References

[1] Stork M., Meindertsma W., Overgaag M., Neils M. (2014). A competitive and efficient lime industry.

[2] Eades J.L., Grim R.E. (1966). A quick test to determine lime requirements for lime stabilization. Highw. Res. Rec..

[3] Strand A., Korotkova E., Willför S., Hakala J., Lindstedt E. (2017). The use of calcium hydroxide as alkali source in peroxide bleaching of kraft pulp. Nordic Pulp & Paper Research. Journal.

[4] Semerjian L., Ayoub G.M. (2003). High-pH–magnesium coagulation–flocculation in wastewater treatment. Adv. Environ. Res..

[5] Yang K.-H., Cho A.-R., Song J.-K., Nam S.-H. (2012). Hydration products and strength development of calcium hydroxide-based alkali-activated slag mortars. Construct. Build. Mater..

[6] Mehdipour I., Falzone G., La Plante E.C., Simonetti D., Neithalath N., Sant G. (2019). How microstructure and pore moisture affect strength gain in portlandite-enriched composites that mineralize CO_2_. ACS Sustain. Chem. Eng..

[7] Vance K., Falzone G., Pignatelli I., Bauchy M., Balonis M., Sant G. (2015). Direct carbonation of Ca(OH)_2_ using liquid and supercritical CO_2_: implications for carbon-neutral cementation. Ind. Eng. Chem. Res..

[8] Gollsch M., Afflerbach S., Drexler M., Linder M. (2020). Structural integrity of calcium hydroxide granule bulks for thermochemical energy storage. Sol. Energy.

[9] Funayama S., Takasu H., Zamengo M., Kariya J., Kim S.T., Kato Y. (2019). Performance of thermochemical energy storage of a packed bed of calcium hydroxide pellets. Energy Storage.

[10] Piringer H. (2017). Lime shaft kilns. Energy Proc..

[11] Apodaca L.E. (2021).

[12] Belhadj E., Diliberto C., Lecomte A. (2012). Characterization and activation of basic oxygen furnace slag. Cement Concr. Compos..

[13] Piatak N.M., Parsons M.B., Seal R.R. (2015). Characteristics and environmental aspects of slag: a review. Appl. Geochem..

[14] (2019). World Steel Association Life Cycle Inventory study report.

[15] Wang H., Wu J.-J., Zhu X., Liao Q., Zhao L. (2016). Energy–environment–economy evaluations of commercial scale systems for blast furnace slag treatment: dry slag granulation vs. water quenching. Appl. Energy.

[16] Chand S., Paul B., Kumar M. (2016). Sustainable approaches for ld slag waste management in steel industries: a review. Metallurgist.

[17] Ober J.A. (2021).

[18] Tossavainen M., Engstrom F., Yang Q., Menad N., Lidstrom Larsson M., Bjorkman B. (2007). Characteristics of steel slag under different cooling conditions. Waste Manag..

[19] Chen Y.-L., Lin C.-T. (2020). Recycling of basic oxygen furnace slag as a raw material for autoclaved aerated concrete production. Sustainability.

[20] Lu T.-H., Chen Y.-L., Shih P.-H., Chang J.-E. (2018). Use of basic oxygen furnace slag fines in the production of cementitious mortars and the effects on mortar expansion. Construct. Build. Mater..

[21] Vallejo Castaño S., Callagon La Plante E., Shimoda S., Wang B., Neithalath N., Sant G. (2021). Calcination-free production of calcium hydroxide at sub-boiling temperatures. RSC Adv..

[22] Sant G.N., Pilon L.G., Callagon La Plante E.B.C.L., Wang B., Wei Z., Vallejo Castaño S. (2021). Facile, low-energy routes for the production of hydrated calcium and magnesium salts from alkaline industrial wastes. US20210024364A1.

[23] Stahel W.R. (2016). The circular economy. Nature.

[24] Sagastume Gutiérrez A., Van Caneghem J., Cogollos Martínez J.B., Vandecasteele C. (2012). Evaluation of the environmental performance of lime production in Cuba. J. Clean. Prod..

[25] Vermeulen J., Whitten T. (1999).

[26] (2017). AspenONE (Version 10).

[27] Castaño S.V., La Plante E.C., Collin M., Sant G., Pilon L. (2022). A pilot-process for calcium hydroxide production from iron slag by low-temperature precipitation. J. Environ. Chem. Eng..

[28] Loncnar M., van der Sloot H.A., Mladenovič A., Zupančič M., Kobal L., Bukovec P. (2016). Study of the leaching behaviour of ladle slags by means of leaching tests combined with geochemical modelling and mineralogical investigations. J. Hazard Mater..

[29] De Windt L., Chaurand P., Rose J. (2011). Kinetics of steel slag leaching: batch tests and modeling. Waste Manag..

[30] Pan S.-Y., Chiang P.-C., Chen Y.-H., Chen C.-D., Lin H.-Y., Chang E.-E. (2013). Systematic approach to determination of maximum achievable capture capacity via leaching and carbonation processes for alkaline steelmaking wastes in a rotating packed bed. Environ. Sci. Technol..

[31] Dražević E., Košutić K., Freger V. (2014). Permeability and selectivity of reverse osmosis membranes: correlation to swelling revisited. Water Res..

[32] Dach H. (2008). Comparison of nanofiltration and reverse osmosis processes for a selective desalination of brackish water feeds. Université d’Angers.

[33] Lawler W., Wijaya T., Antony A., Leslie G., Le-Clech P. (2011).

[34] Mulder M. (1991).

[35] Oren Y.S., Biesheuvel P.M. (2018). Theory of ion and water transport in reverse-osmosis membranes. Phys. Rev. Appl..

[36] Bergman T.L., Lavine A.S., Incropera F.P., Dewitt D.P. (2011).

[37] WAVE Software for Water Treatment Plant Design. https://www.dupont.com/water/resources/design-software.html (accessed July 17, 2020).

[38] Filmtec BW30-400 High Rejection Brackish Water RO Membrane Element 10500 GPD. Fresh Water Systems n.d. https://www.freshwatersystems.com/products/filmtec-bw30-400-high-rejection-brackish-water-ro-membrane-element-10500-gpd (accessed July 9, 2020).

[39] DOW Filmtec BW30-400 RO Membrane 10500 GPD n.d. http://www.filterwater.com/p-741-dow-filmtec-bw30-400-ro-membrane-10500-gpd.aspx (accessed July 9, 2020).

[40] Filmtec BW30-400/34i High Rejection Brackish Water RO Membrane Element With Interlocking End Caps, 10500 GPD. Fresh Water Systems n.d. https://www.freshwatersystems.com/products/filmtec-bw30-400-34i-high-rejection-brackish-water-ro-membrane-element-with-interlocking-end-caps-10500-gpd (accessed July 9, 2020).

[41] Yun T.I., Gabelich C.J., Cox M.R., Mofidi A.A., Lesan R. (2006). Reducing costs for large-scale desalting plants using large-diameter, reverse osmosis membranes. Desalination.

[42] (2008). Historical inflation rates: 1914-2020. US Inflation Calculator.

[43] Prices and factors affecting prices - U.S. Energy Information Administration (EIA). https://www.eia.gov/energyexplained/electricity/prices-and-factors-affecting-prices.php (accessed July 9, 2020).

[44] Choi Y., Cho H., Shin Y., Jang Y., Lee S. (2015). Economic evaluation of a hybrid desalination system combining forward and reverse osmosis. Membranes.

[45] (2006). Green Delta.

[46] Skone T.J. National energy technology laboratory. Carbon dioxide utilization – NETL CO2U LCA guidance toolkit. https://www.netl.doe.gov/energy-analysis/details.

[47] National Renewable Energy Laboratory (2012). U.S. Life cycle inventory database. https://www.nrel.gov/lci/.

[48] Bond F.C. (1961). Crushing and grinding calculations. Br. Chem. Eng..

[49] Weiss N.L. (1985).

[50] Kirchofer A., Becker A., Brandt A., Wilcox J. (2013). CO_2_ mitigation potential of mineral carbonation with industrial alkalinity sources in the United States. Environ. Sci. Technol..

[51] Bonenfant D., Kharoune L., Sauvé S., Hausler R., Niquette P., Mimeault M. (2008). CO_2_ sequestration potential of steel slags at ambient pressure and temperature. Ind. Eng. Chem. Res..

[52] Curry K.C., USGS (2020).

[53] NATCARB CO2 Sources v1501 (Archived) - EDX. NETL’s Energy Data Exchange n.d. https://edx.netl.doe.gov/dataset/natcarb-co2sources-v1501-archived (accessed June 18, 2021).

[54] Gingerich D.B., Mauter M.S. (2015). Quantity, quality, and availability of waste heat from United States thermal power generation. Environ. Sci. Technol..

[55] U.S. Energy Information Agency Form EIA-923 detailed data. https://www.eia.gov/electricity/data/eia923/.

[56] U.S. Energy Information Agency Form EIA-860 detailed data. https://www.eia.gov/electricity/data/eia860/.

[57] (2020). MATLAB (Version 2020).

[58] QGIS Geographic Information System (2018). http://qgis.org.

[59] Kirchofer A., Becker A., Brandt A., Wilcox J. (2013). CO_2_ mitigation potential of mineral carbonation with industrial alkalinity sources in the United States. Environ. Sci. Technol..

[60] (2021). Mineral Commodity Summaries.

[61] National Slag Association - Slag availability. National Slag Association n.d. http://nationalslag.org/?s=&category=&location=&a=true&count=20&orderby=title&order=ASC (accessed August 30, 2021).

[62] Kuroki T., Kabeya K., Makino K., Kajihara T., Kaibe H., Hachiuma H. (2014). Thermoelectric generation using waste heat in steel works. Journal of Elec Materi.

