# Supplementary material for: Analyzing the upscaling potential and geospatial siting of calcination-free calcium hydroxide production in the United States
Source: Heliyon. 2024 Jun 4;10(12):e32426. doi: 10.1016/j.heliyon.2024.e32426 (PMC11226802; doi:10.1016/j.heliyon.2024.e32426)
Supplement: Multimedia component 1 [file mmc1.docx]

***Supporting Information***

**Analyzing the upscaling potential and geospatial siting of calcination-free Ca(OH)_2_ production in the United States**

| 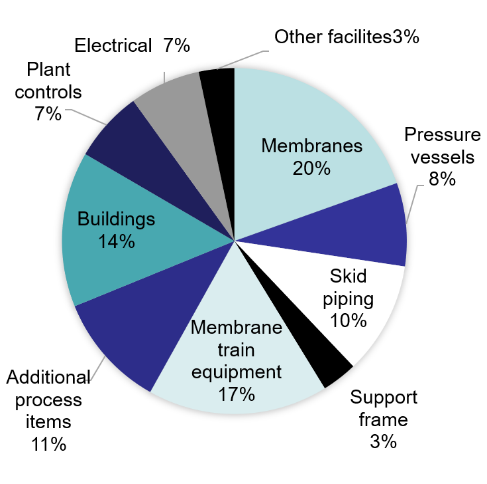  **Figure S1.** The breakdown of capital costs for a typical commercial scale RO installation. |
| --- |
